# Changes in the liver transcriptome and physiological parameters of Japanese Black steers during the fattening period

**DOI:** 10.1038/s41598-022-08057-8

**Published:** 2022-03-07

**Authors:** Minji Kim, Tatsunori Masaki, Kentaro Ikuta, Eiji Iwamoto, Yoshinobu Uemoto, Fuminori Terada, Sanggun Roh

**Affiliations:** 1 Graduate School of Agricultural Science, Tohoku University, Sendai, 980-8572 Japan; 2 Hyogo Prefectural Technology Center of Agriculture, Forestry and Fisheries, Kasai, Hyogo 679-0198 Japan; 3 Awaji Agricultural Technology Center, Minami-Awaji, Hyogo 656-0442 Japan; 4 Present Address: National Institute of Livestock and Grassland Science, National Agriculture and Food Research Organization, Ikenodai, Tsukuba, 305-0901 Japan

**Keywords:** Metabolism, Animal physiology

## Abstract

We investigated the physiological changes during the fattening period and production characteristics in Japanese Black steers bred and raised using the typical feeding system in Japan. Here, 21 Japanese Black steers aged 12 months were used, with experimental period divided into early (12–14 months of age), middle (15–22 months), and late fattening phases (23–30 months). The liver transcriptome, blood metabolites, hormones, and rumen fermentation characteristics were analyzed. Blood triglyceride and non-esterified fatty acid concentrations increased, whereas blood ketone levels decreased, with fattening phases. Blood insulin increased with fattening phases and was positively correlated with carcass weight and marbling in late fattening phases. Rumen fermentation characteristics showed high propionate levels and low butyrate levels in late fattening phases, likely due to increased energy intake. Genes related to glucose metabolism, such as *SESN3, INSR, LEPR, and FOXO3*, were down-regulated in late fattening phases. Genes related to lipid metabolism, such as *FABP4,* were up-regulated, whereas *FADS1* and *FADS2* were down-regulated. These findings suggest that the physiological changes resulted from changes in the energy content and composition of diets. Liver metabolism changed with changes in fat metabolism. Insulin was strongly associated with physiological changes and productivity in Japanese Black cattle.

## Introduction

Japanese Black cattle breed is well-known for producing premium beef with high levels of intramuscular fat (marbling), and has been improved by genetic selection in the last half century. It has genetic characteristics that allow more intramuscular fat accumulation compared to other beef cattle. Therefore, Japan has a special feeding management system that includes using a large amount of concentrate and controlling the vitamin A levels in the feed and cattle blood to improve the marbling in beef^1^. Based on these differences, the Japanese Black is likely to have differences in physiology compared to other beef cattle. However, previous studies of Japanese Black cattle focused on its genetic features and improvement of management strategies for producing high-quality beef. Therefore, there is a lack of research on the physiological features of this cattle breed. The physiological characteristics in of crossbred black cattle raised outside Japan have been investigated in several studies. Connolly et al.^2^ reported the relationship between blood metabolome and fat accumulation in Wagyu crossbred steers and suggested that the relationship is influenced by days in a feedlot and the growth development status. Another study reported that Japanese Black × Angus steers had higher triacylglycerol content and enzymes activities involved in de novo lipogenesis compare to Limousin steers^3^. This study also suggested that physiological characteristics differ according to breed, even in beef cattle produced for meat production. However, even if the genetic background is partially similar, the physiological features of Japanese Black cattle likely differ from those of crossbred cattle because their rearing environment and feeding management differ. Therefore, it is necessary to investigate the physiological features of Japanese Black cattle raised by feeding management developed according to the livestock practices in Japan.

The present study focused on the liver transcriptome to investigate the physiological features of Japanese Black cattle. In ruminants, the liver regulates the overall energy balance by synthesizing glucose with propionic acid absorbed from the rumen^4^ and regulates fat metabolism through fat oxidation and accumulation^5^. Recent studies have shown that liver function is related to livestock productivity, such as meat and milk production. Brown et al.^6^ reported that the carcass weight and rib eye area were lower in cattle with liver abnormalities than in those with normal liver function. The study also reported that liver function abnormalities caused a significant economic liability to the cattle feeding and beef processing industries. Therefore, this study aimed to investigate the physiological features associated with the fattening period and production characteristics in Japanese Black steers bred and raised in Japan. To comprehensively examine the metabolic status of black steers according to the fattening period, we collected various physiological parameters, including the liver transcriptome, blood metabolites, hormones, and rumen fermentation.

## Results

### Blood metabolome and rumen fermentation profiles

Table 1 shows the concentrations of blood metabolites and hormones in the early (T1), middle (T2), and late (T3) fattening phases in Japanese Black cattle. The concentrations of several blood metabolites increased significantly (*P* < 0.05) during the middle and late fattening phases, including creatinine, triglyceride, total cholesterol, non-esterified fatty acids (NEFA), phospholipid, alanine aminotransferase (ALT), gamma-glutamyl transferase (γ-GTP), and lactate dehydrogenase (LD), whereas those of blood glucose, alkaline phosphatase (ALP), and β-hydroxybutyric acid (BHBA) decreased during the late fattening phase (*P* < 0.05). The concentrations of insulin and cortisol increased significantly (*P* < 0.05) as fattening progressed, whereas that of the insulin-like growth factor 1 (IGF-I) decreased (*P* < 0.05). Supplementary Table S1 online lists the concentrations of blood amino acids during each fattening period in Japanese Black Steers. Threonine, glycine, alanine, valine, methionine, isoleucine, arginine, and hydroxyproline levels were higher in T1 than in T2 and T3, whereas glutamine, cysteine, tyrosine, and 1-methylhistidine levels were higher in T3 (*P* < 0.05). Taurine, α-amino adipic acid, and ornithine concentrations increased in T2 and then sharply decreased in T3 (*P* < 0.05). In rumen fermentation samples, the pH and NH_3_ levels were significantly lower (*P* < 0.01) in T3 than in T1 and T2 (Supplementary Table S2 online). The percent of propionate increased significantly (*P* < 0.01) with the fattening periods, whereas the acetate and butyrate levels decreased (*P* < 0.01 and *P* < 0.1, respectively).

**Table 1:** Concentrations of blood metabolites and hormones during the early, middle, and late fattening phases cattle in the early (T1; 13 months of age), middle (T2; 20 months of age), and late fattening phases (T3; 28 months of age).

Variable	Period			SEM	P-value
	T1	T2	T3		
**Blood metabolites**					
Total protein (g/dL)	6.48	6.59	6.61	0.05	0.21
Albumin (g/dL)	3.53b	3.67a	3.57b	0.02	< 0.01
BUN (mg/dL)	14.68a	15.36a	9.85b	0.42	< 0.01
Creatinine (mg/dL)	1.01b	1.21a	1.29a	0.02	< 0.01
Total cholesterol (mg/dL)	96.24b	130.67a	99.52b	3.04	< 0.01
Triglyceride (mg/dL)	12.48b	14.57ab	16.05a	0.48	< 0.01
NEFA (mEq/L)	0.08b	0.09b	0.11a	< 0.01	< 0.01
Phospholipid (mg/dL)	90.9b	117.24a	91.57b	2.40	< 0.01
Glucose (mg/dL)	71.62a	66.33b	67.9b	0.04	< 0.01
ALP (U/L)	319.81a	221.43b	210.14b	8.99	< 0.01
AST (U/L)	71.62	66.33	67.90	2.16	0.35
ALT (U/L)	19.14a	17.00b	21.48a	0.48	< 0.01
γ-GTP (U/L)	20.86b	29.43a	22.33b	0.82	< 0.01
LD (U/L)	943.43c	1114.19b	1297.86a	26.45	< 0.01
CK (U/L)	144.71	137.33	147.71	5.81	0.72
Acetate (μmol/L)	21.62a	8.62b	8.52b	2.26	< 0.01
BHBA (μmol/L)	717.52a	531.71b	500.33b	24.09	< 0.01
Total ketone body (μmol/L)	753.10a	540.10b	508.38b	26.12	< 0.01
**Hormones**					
Insulin (ng/mL)	2.22b	3.82a	4.08a	0.28	< 0.01
IGF-I (ng/mL)	206.36a	139.18b	149.48b	6.51	< 0.01
Cortisol (ng/mL)	4.70b	9.62b	21.61a	1.68	< 0.01

Values indicate mean and means in the same row with different alphabets differ significantly (P < 0.05).

SEM: standard error of the mean, BUN: blood urea nitrogen, NEFA: non-esterified fatty acid, ALP: alkaline phosphatase, AST: aspartate aminotransferase, ALT: alanine aminotransferase, γ-GTP: gamma(γ)-glutamyl transferase, LD: lactate dehydrogenase, CK: creatine kinase, BHBA: β-hydroxybutyric acid, IGF-I: insulin-like growth factor 1.

**Figure 1:**
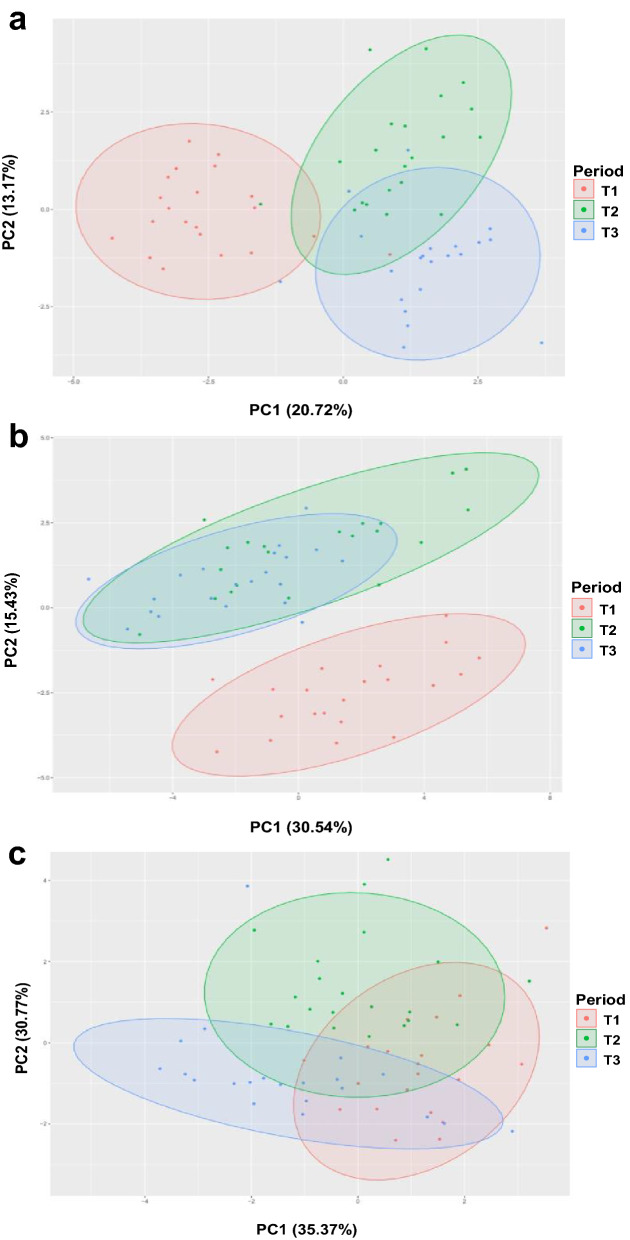
Principal component analysis of physiological parameter of 21 Japanese black cattle, showing the PC1 vs PC2 values. The fattening phases are coloured differently for each data point. T1: early fattening phases (13 months of age), T2: middle fattening phases (20 months of age), T3: late fattening phases (28 months of age). (**a**) Principal component analysis of 18 blood metabolites and 3 hormones. (**b**) Principal component analysis of 29 blood amino acid. (**c**) Principal component analysis of 9 rumen fermentation characteristics.

Figure 1a shows the principal component analysis (PCA) score plot of the 18 blood metabolites and 3 hormones of Japanese Black cattle, with each data point colored according to the fattening phase. PC1 and PC2 explained 20.72% and 13.17% of the total variance, respectively, and the clustering patterns suggested differences in blood metabolite profiles among the fattening phases. Figure 1b shows the PCA score plot for the 29 blood amino acids of Japanese Black cattle, where PC1 and PC2 explained 30.54% and 15.43% of the total variance, respectively. Blood amino acids in T1 were different from those in T2 and T3. However, in the PCA plot of rumen fermentation characteristics (Fig. 1c), there was no distinct clustering according to the fattening period compared to the blood composition.

### Gene expression analysis by RNA-Seq

RNA-sequencing (RNA-seq) of the liver tissue generated 2,965,384,118 reads and 299,503,795,918 base pairs during the entire experimental period. The average reads of each period were 49.60, 49.86, and 51.37 million in T1 (n = 20), T2 (n = 18), and T3 (n = 19), respectively, and the clean read rate was 95.39%, 95.83%, and 95.84% in each period, respectively (Supplementary Tables S3 and S4 online). In total, 19,063, 19,649, and 19,343 expressed genes were detected in the liver of black steers in T1, T2, and T3, respectively (Supplementary Fig. S1 online). Of these, 18,317 genes were expressed during all fattening phases; 298, 197, and 482 genes were commonly identified between pairs of groups (T1 vs T2, T1 vs T3, and T2 vs T3, respectively); and 251, 552, and 347 genes were detected exclusively in T1, T2, and T3, respectively (Supplementary Fig. S1 online).

**Figure 2:**
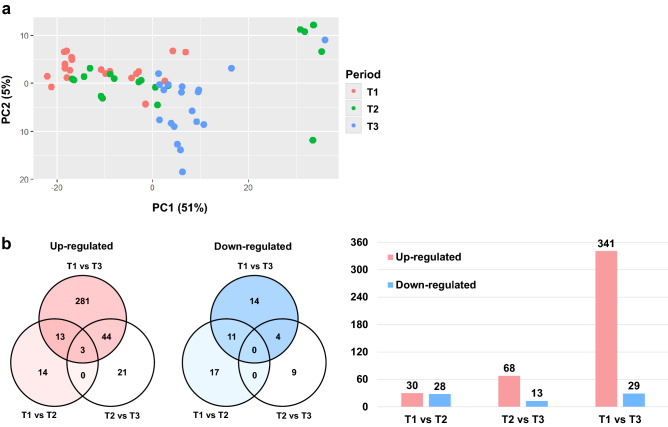
Summary of differentially expressed genes of Japanese black cattle in the early (T1; 13 months of age), middle (T2; 20 months of age), and late fattening phases (T3; 28 months of age). (**a**) Principal component analysis of identified and annotated genes of Japanese black cattle, showing the PC1 vs PC2 plot with each data point colored according to fattening phase. (**b**) Venn diagram and bar graph of the number of upregulated and downregulated differentially expressed genes between the fattening phases. Significant differentially expressed genes were defined with FDR < 0.05, |log_2_ Fold Change|> 1.5, and base mean > 30 in this study.

Figure 2a shows the PCA plot (with 2 outliers removed from T2) of gene expressions in individual samples. PC1 and PC2 explained 51% and 5% of the variance within the dataset of annotated genes, and the plot showed clustering within each fattening phase, particularly in T1 and T3.

To investigate differentially expressed genes (DEGs) in the liver tissues between pair of fattening phases (T1 vs T2, T1 vs T3, and T2 vs T3), we defined hepatic genes as differentially expressed according to the following criteria: a ≥ 1.5 log_2_ fold change in expression level, base mean > 30, and false discovery rate (FDR) of < 0.05. Volcano plots were prepared to visualize the DEGs between 2 phases, with red points marking the genes with significantly increased or decreased expression (Supplementary Fig. S2 online). In total, 509 DEGs were identified between any 2 periods, with 58 (30 upregulated and 28 downregulated), 370 (341 upregulated and 29 downregulated), and 81 (68 upregulated and 13 downregulated) DEGs between T1 vs T2, T1 vs T3, and T2 vs T3, respectively (Fig. 2b). Additionally, 4 Japanese Black cattle with high vs low carcass weight and beef marbling score (BMS) were identified, and their hepatic transcriptomes were analyzed (Table 2, Supplementary Tables 5 and 6 online). In total, 14 and 5 DEGs were found in the comparisons of groups with different carcass weights (high vs low) and BMS (high vs low), respectively.

**Table 2:** Mean carcass traits in carcass weight and BMS groups.

Variable	Carcass weight group				BMS group			
	High	Low	SEM	P-value	High	Low	SEM	P-value
Live weight (kg)	782.50	698.00	18.30	< 0.01	770.50	747.50	11.80	0.39
Growth rate (kg/d)	0.84	0.75	0.02	0.02	0.83	0.81	0.01	0.62
Carcass weight (kg)	491.50	417.25	14.89	< 0.01	482.00	452.75	8.79	0.10
Eye muscle area (cm^2^)	59.25	50.00	2.59	0.07	57.25	58.50	2.59	0.83
Rib thickness (cm)	8.38	6.98	0.33	0.02	8.23	7.70	0.23	0.30
Subcutaneous fat (cm)	3.50	2.28	0.26	< 0.01	3.38	2.33	0.27	0.05
BMS	9.50	7.50	0.63	0.12	10.00	6.50	0.70	< 0.01

High: group of cattle with high productivity (n = 4), Low: group of cattle with low productivity (n = 4). Values indicate the mean. SEM: standard error of the mean, BMS: beef marbling score (Japanese standards ranged from 1, which contains no visible marbling, up to 12, which is heavily marbled).

### Gene ontology analysis of DEGs

Gene ontology (GO) analysis was conducted by running queries for each DEG against the GO database, which provided information on relevant biological processes (BP), cellular components (CC), and molecular functions (MF) associated with the DEGs. GO and pathway analysis of the DEGs in T1 and T3 showed the greatest difference in gene expression. The DEGs between T1 and T3 were mainly involved in energy and lipid metabolism processes, such as glucose homeostasis, cholesterol biosynthetic process, phospholipid homeostasis, triglyceride biosynthetic process, and fatty acid metabolic process (Tables 3 and 4, Supplementary Tables 7 and 8 online). The CC- and MF-related genes were enriched in substance transport processes, including calcium ion binding, transporter activity, and voltage-gated chloride channel activity (Table 3, Supplementary Tables 7 and 8 online).

**Table 3:** Gene ontology of differentially expressed genes in early fattening phases (13 months of age) vs late fattening phases (28 months of age).

Ontology	GO term ID	Description	No. of DEGs	P-value
Biological processes	GO:0008152	Metabolic process	7	0.01
	GO:0001525	Angiogenesis	7	0.03
	GO:0042593	Glucose homeostasis	6	0.02
	GO:0030335	Positive regulation of cell migration	6	0.07
	GO:0006695	Cholesterol biosynthetic process	5	< 0.01
	GO:0032922	Circadian regulation of gene expression	5	0.01
	GO:0060395	Smad protein signal transduction	5	0.02
	GO:0006469	Negative regulation of protein kinase activity	5	0.04
	GO:0035264	Multicellular organism growth	5	0.04
	GO:0006491	N-glycan processing	4	0.01
	GO:0018107	Peptidyl-threonine phosphorylation	4	0.01
	GO:0006629	Lipid metabolic process	4	0.07
	GO:0051897	Positive regulation of protein kinase B signaling	4	0.09
	GO:0006874	Cellular calcium ion homeostasis	4	0.10
	GO:0055091	Phospholipid homeostasis	3	< 0.01
	GO:0019432	Triglyceride biosynthetic process	3	0.01
	GO:0003222	Ventricular trabecula myocardium morphogenesis	3	0.02
	GO:0006631	Fatty acid metabolic process	3	0.07
	GO:0001568	Blood vessel development	3	0.09
	GO:0006814	Sodium ion transport	3	0.09
Cellular composition	GO:0016021	Integral component of membrane	107	< 0.01
	GO:0070062	Extracellular exosome	57	< 0.01
	GO:0005887	Integral component of plasma membrane	26	0.01
	GO:0005783	Endoplasmic reticulum	20	< 0.01
	GO:0005794	Golgi apparatus	17	0.03
Molecular functions	GO:0005509	Calcium ion binding	23	< 0.01
	GO:0004571	Mannosyl-oligosaccharide 1,2-alpha-mannosidase activity	3	0.01
	GO:0015269	Calcium-activated potassium channel activity	3	0.02
	GO:0005215	Transporter activity	6	0.03
	GO:0004008	Copper-exporting atpase activity	2	0.03

GO: gene ontology, DEGs: differentially expressed genes.

**Table 4:** Gene ontology of differentially expressed genes related to lipid and energy metabolism in early fattening phases (13 months of age) vs late fattening phases (28 months of age).

GO term ID	Description	No. of DEGs	P-value	Up-regulated genes	Down-regulated genes
GO:0008152	Metabolic process	7	0.01	*LPGAT1, FASN, EDEM1, IDS, DIP2C, PIGG*	*GSTA3*
GO:0042593	Glucose homeostasis	6	0.02	*SESN3, HNF4A, INSR, LEPR, PTPN11, FOXO3*	
GO:0006695	Cholesterol biosynthetic process	5	0.00	*NPC1L1, DHCR24, HMGCR, DHCR7, HSD17B7*	
GO:0006629	Lipid metabolic process	4	0.07	*FADS2, ACOX1, HNF4A, FADS1*	
GO:0055091	Phospholipid homeostasis	3	0.00	*ABCA1, GPAM, HNF4A*	
GO:0019432	Triglyceride biosynthetic process	3	0.01	*GPAM, LPIN1, LPIN2*	
GO:0006631	Fatty acid metabolic process	3	0.07	*GPAM*	*FABP4, SNCA*

GO: gene ontology, DEGs: differentially expressed genes.

### Pathway analysis of DEGs

We used the KEGG database (http://www.kegg.jp/kegg/kegg1.html) to determine significant pathways involving the DEGs between the early and late fattening phases. In total, 30 pathways were significantly enriched for the identified DEGs (*P* < 0.05 and *P* < 0.1; Supplementary Table 9 online). Among these, the top 10 DEGs were mainly involved in lipid metabolism and signaling pathways, including metabolic pathways, glycerolipid metabolism, bile secretion, glycerophospholipid metabolism, AMPK signaling pathway, cGMP-PKG signaling pathway, calcium signaling pathway, fatty acid metabolism, PPAR signaling pathway, and vascular smooth muscle contraction. Moreover, 41 genes were involved in the metabolic pathway, including 40 up-regulated genes and 1 down-regulated gene.

### Association between physiological parameters and carcass traits

Supplementary Table S10 online summarizes the carcass traits after slaughter. The average carcass weight and eye muscle area were 458.8 kg and 55.9 cm^2^, respectively. The BMS was 6–11 (average, 8.3). The Japan black cattle used here had lower carcass weight and eye muscle area, and similar in rib thickness and subcutaneous fat thickness, compared to the national average in Japan in 2019^7^ which may be considered as characteristic of Tajima cattle (i.e., the Wagyu breed produced in Hyogo Prefecture, Japan) used in this experiment.

Table 5 lists the feed composition during the T1, T2, and T3. Table 6 lists the correlation coefficients between carcass traits and physiological parameters in T1. Blood triglyceride was positively (*P* < 0.05) correlated with rib eye area and BMS. ALT levels were positively correlated (*P* < 0.05) with live weight, growth rate, carcass weight, rib thickness, and subcutaneous fat. The ALP and γ-GTP were not significantly correlated with carcass trait. IGF-1 showed a positive trend with carcass trait in T1. Taurine, leucine, and phenylalanine levels were negatively correlated (*P* < 0.05) with BMS. Aspartic acid level was positively correlated with rib thickness and BMS (*P* < 0.05), and 1-methylhistidine and hydroxyproline levels were positively correlated with rib thickness and subcutaneous fat (*P* < 0.05). In rumen fermentation samples, pH was negatively correlated with BMS and NH_3_ was positively correlated with eye muscle area (*P* < 0.05) (Table 6).

**Table 5:** Mean growth performance and nutrient composition in Japanese black cattle during the early, middle, and late fattening phases.

Variable	Period		
	T1	T2	T3
**Growth performance**			
Average body weight (kg)	369.8 (337.7–400.7)	522.6 (478.9–584.3)	686.9 (607.0–757.0)
Average dairy gain (kg/day)	0.86 (0.45–1.07)	0.83 (0.61–1.04)	0.61 (0.49–0.80)
**Feed intake**			
Concentrate (kg/day)	5.41 (4.13–5.61)	7.31 (5.96–8.30)	7.44 (4.67–8.96)
Rice straw (kg/day)	1.93 (1.21–2.70)	1.13 (0.72–1.61)	0.74 (0.49–0.99)
Kraft pulp feed (kg/day)	0.72 (0.00–1.54)	0.45 (0.00–0.92)	0.29 (0.00–0.60)
Dry matter (kg/day)	6.98 (5.67–7.27)	7.72 (6.52–8.65)	7.36 (4.89–8.69)
Total digestible nutrients (kg/day)	4.95 (3.94–5.28)	5.95 (5.00–6.69)	5.84 (3.84–6.89)
Crude protein (kg/day)	0.95 (0.74–1.02)	0.93 (0.75–1.07)	0.93 (0.59–1.12)

T1: early fattening phase (13 months of age), T2: middle fattening phases (20 months of age), T3: late fattening phases (28 months of age); The cattle were fed concentrates as follows: T1: steam-flaked corn 42%, wheat bran 27%, corn starch 10%, soybean meal 12%, soybean hull 6%, salt 1% (TDN 71.2%, CP 15.9%); T2: steam-flacked corn 42%, barley 14%, wheat bran 21%, corn starch 5%, soybean meal 10%, soybean hull 6%, salt 1% (TDN 72.5%, CP 14.4%); T3: steam-flacked corn 44%, barley 25%, wheat bran 14%, soybean meal 5%, soybean hull 10%, salt 1% (TDN 72.8%, CP 12.0%). The rice straw intakes were as follows: DM 87.8%, TDN 37.7%, CP 4.7%. The kraft pulp feed intakes were as follows: DM 76.9%, TDN 51.1%, CP 0.3%

**Table 6:**
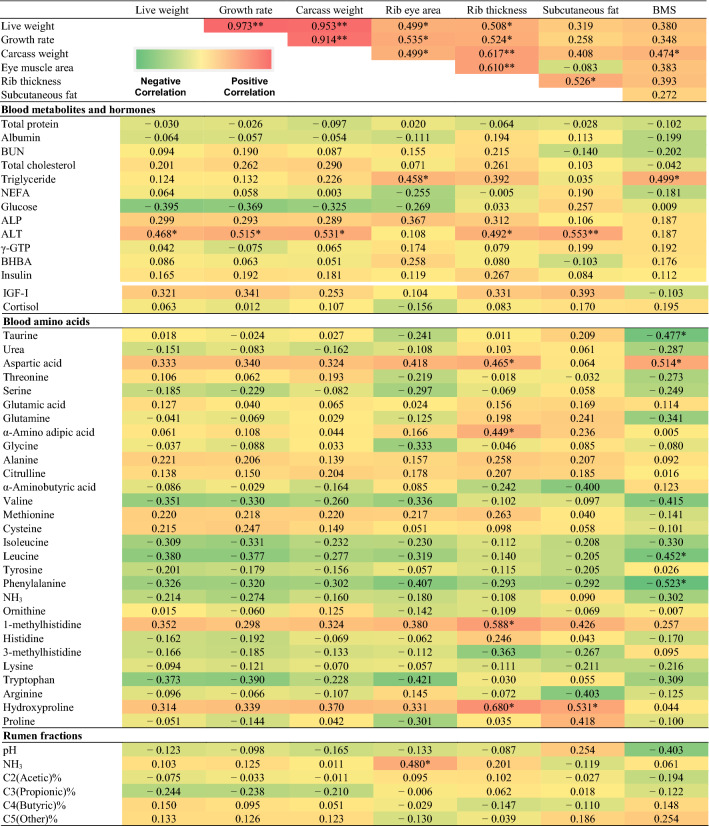
Heat map illustrating the correlations between physiological parameters and carcass traits in the early fattening period (13 months of age).

BMS: beef marbling score, BUN: blood urea nitrogen, NEFA: non-esterified fatty acid, ALP: alkaline phosphatase, AST: aspartate aminotransferase, ALT: alanine aminotransferase, γ-GTP: gamma(γ)-glutamyl transferase, LD: lactate dehydrogenase, CK: creatine kinase, BHBA: β-hydroxybutyric acid, IGF-I: insulin-like growth factor 1. ** and * indicate P < 0.01 and P < 0.05, respectively.

Table 7 shows the correlation coefficients between carcass traits and physiological parameters in T3. Blood albumin was negatively correlated with carcass weight and BMS (*P* < 0.05), but BUN exhibited a positive trend with carcass weight. Blood glucose was positively correlated with subcutaneous fat (*P* < 0.05). ALP was significantly negatively correlated with growth rate (*P* < 0.05) and also with carcass weight, rib thickness, and subcutaneous fat. Ketones were positively correlated with all carcass traits. Unlike in the T1 group, blood insulin in T3 was positively correlated with carcass traits (*P* < 0.05). IGF-I was negatively correlated with BMS, and cortisol level showed no correlation with carcass traits. Blood taurine and urea showed positive correlations with growth rate and carcass weight. Rib thickness was positively correlated with α-amino adipic acid and negatively correlated with glycine, arginine, and hydroxyproline levels (*P* < 0.05). BMS was negatively correlated with cysteine and proline levels (*P* < 0.05). In rumen fermentation samples, there was no significant correlation in the T3 group (Table 7).

**Table 7:**
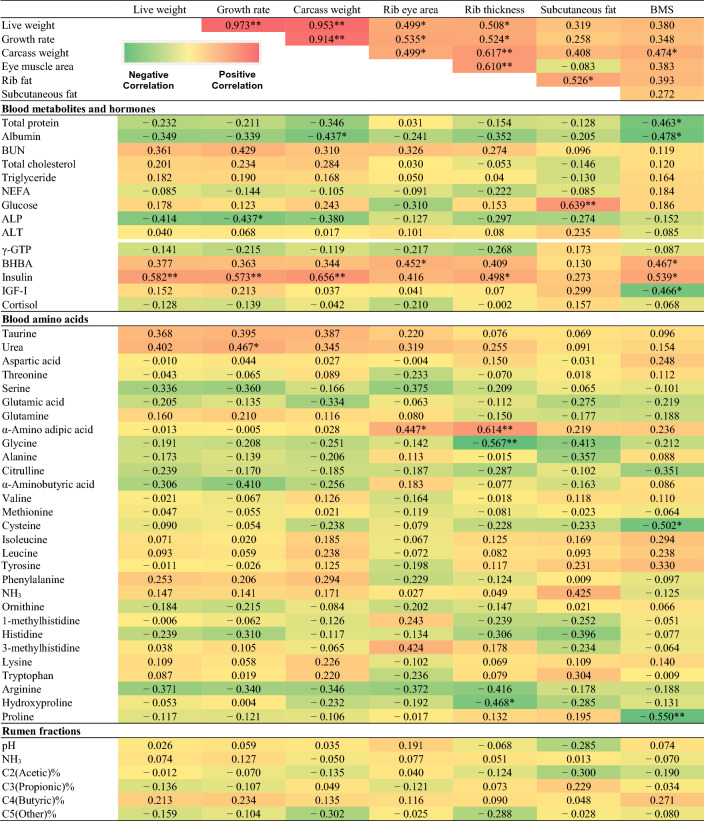
Heat map illustrating the correlations between physiological parameters and carcass traits in the late fattening period (28 months of age).

BMS: beef marbling score, BUN: blood urea nitrogen, NEFA: non-esterified fatty acid, ALP: alkaline phosphatase, AST: aspartate aminotransferase, ALT: alanine aminotransferase, γ-GTP: gamma(γ)-glutamyl transferase, LD: lactate dehydrogenase, CK: creatine kinase, BHBA: β-hydroxybutyric acid, IGF-I: insulin-like growth factor 1. ** and * indicate *P* < 0.01 and *P* < 0.05, respectively.

## Discussion

We comprehensively analyzed the metabolic status of Japanese Black steers according to fattening phase, including various physiological parameters such as the liver transcriptome, blood metabolites, hormones, and rumen fermentation characteristics. The major physiological changes associated with fattening period and productivity are discussed below.

### Changes in physiological parameters in Japanese Black steer according to fattening phase

We reported the changes in metabolite profiles, hormones, and rumen fermentation characteristics during the fattening period in Japanese Black steer bred and raised in Japan. The triglyceride and NEFA concentrations increased with fattening phase due to the changes in the feed, including higher amounts of concentrated diet and increased dry matter intake. Increasing amounts of the concentrated diet from T1 to T2 elevated the total cholesterol, phospholipids, glucose, and γ-GTP levels in T2; however, the levels of these metabolites in T3 were similar to those in T1. Japanese Black cattle are fed large amounts of high-energy concentrate diets in the middle and late fattening phases to improve their growth performance and productivity. A study on Yellow breed × Simmental cattle showed that lipid metabolism-related blood metabolites (triglycerides, cholesterol, and high-density and low-density lipoprotein) were significantly higher in the high-energy group (6.64 MJ/kg) than in the low-energy group (5.98 MJ/kg), and that blood insulin levels increased with increasing energy levels^8^. Blood glucose and insulin levels also increase significantly with high-starch diets in Angus and Angus × Simmental cattle^9^. In ruminants, concentrate diets are a major source of glucose, either through increased production of rumen propionate (a gluconeogenic precursor), or—to a smaller extent—through glucose absorption from rumen-bypassed diets^10^. Glucose synthesized via gluconeogenesis is the primary energy source in ruminants^11^, and excess glucose is converted into fatty acids that circulate to other parts of the body and are stored as fat in adipose tissue^12^. Accordingly, the high-energy feeding boosts rumen propionate production and liver gluconeogenesis, and may explain the increased concentrations of blood lipid metabolites during the T2 and T3. In cattle, NEFAs increase due to energy deficiency^13^ or pathological problems such as ketosis and fatty liver^14^. In a state of negative energy balance, NEFAs are produced via lipolysis from triglycerides stored in adipose tissue and transported to organs and tissues^15^. In the present study, high blood levels of NEFAs in the middle and late fattening phases were likely due to relative increases in body fat reserves as fattening progressed, rather than energy deficiency. Samra et al.^16^ reported that cortisol—an indicator of stress response—promotes lipolysis, thus increasing blood NEFA concentrations. The increased cortisol levels during the fattening period in this study may have promoted lipolysis, resulting in increased concentrations of NEFA in the blood. The blood insulin level depends on age, weight, diets, and feeding management^17^, and is generally known to increase as fattening progresses. Ruminants are less sensitive to insulin compared to monogastric animals, as insulin secretion is mainly stimulated by VFAs^18,19^ and the expression of glucose transporter type 4, which is an insulin dependent glucose transporter, is very low in cells such as adipocyte^20^. Propionate is a strong stimulator of insulin secretion in sheep^21^. The increase in insulin secretion from T1 to T3 may be related to the increase in rumen propionate during the fattening period, and may have facilitated the intracellular influx of glucose, thus reducing blood glucose levels. Generally, BHB and other ketone bodies increase during energy deficit and are produced as metabolites in the lipid oxidation process of NEFAs in the liver. Here, the ketone concentrations decreased significantly in the middle and late fattening phases. Particularly, the decrease in BHB levels was likely due to the decreased levels of its precursor, rumen butyrate, during the T2 and T3. To evaluate the liver function in Japanese Black steers, we analyzed the blood levels of ALP, AST, ALT, and γ-GTP. These metabolites are present in hepatocytes and released into the blood stream when hepatocytes are damaged by stimulation such as mold toxins and long-term high-energy feeding. Blood ALP—mainly released from the liver and bones—is related to liver dysfunction and bone development in beef cattle^22,23^. In growing animals, blood ALP is mainly released from bone tissue; the main tissue is changed from bone to the liver and the overall release decreases with growth^24,25^. In Japanese Black calves, blood ALP concentrations are significantly lower in the late growing stage than in the early growing stage, likely due to the slower rate of bone growth with increasing age^24^. In accordance with the findings of the previous study, our results showed that the blood ALP concentration decreased with fattening phases, suggesting that blood ALP concentrations decrease with age unless there is a severe abnormality in liver function. The blood γ-GTP is mainly released from the liver and bile duct and reflects chronic liver function because changes in its blood concentration occur at a slower rate than those of AST and ALT^26^. Blood γ-GTP concentration is positively correlated with BUN levels in Japanese Black cattle during the fattening period. Similar to the results of the previous study, our results showed that the blood γ-GTP levels were comparatively higher in T2 and T3 than in T1, and the reduction was likely caused by increased feed intake in T2 and T3. The above 4 enzymes increased or decreased slightly with the fattening period, but all were within the normal range.

Amino acids are not only the organic components of proteins, but are also involved in various physiological mechanisms, such as glucose synthesis via gluconeogenesis, fatty acid synthesis, and hormone regulation^27^. Amino acid metabolism is closely related to liver function because the absorption and transformation of amino acids occur in the liver. Thus, the blood concentrations of amino acids can be used to determine the nutritional and metabolic conditions of animals. Here, 22 of the 29 blood amino acids showed differences in concentration between fattening phases, and 13 decreased during the late fattening phase. Connolly et al.^2^ also reported that the progression of the feeding period in the feedlot was associated with decreasing blood concentrations of amino acids such as proline, leucine, isoleucine, histidine, whereas aspartate, and glutamic acid (a typical gluconeogenic acid) increased in Wagyu crossbred steers.

### Liver transcriptome changes in Japanese Black cattle according to fattening period

We conducted the GO pathway analysis for enriched DEGs to define the functional roles of DEGs according to fattening period. Here, we focused on comparisons between early and late fattening phases, which showed the greatest differences in gene expression in the DEG analysis. The DEGs were significantly enriched in metabolic processes such as glucose and lipid metabolic process.

The expression of genes related to glucose metabolism—such as sestrin3 (*SESN3*), insulin receptor (*INSR*), leptin receptor (*LEPR*), forkhead box O1 (*FOXO3*)—was decreased in late fattening phases**.** LEPR is a liver-specific leptin receptor and regulates energy balance. Hepatic *LEPR* expression is positively correlated with plasma leptin concentration^28^; serum leptin concentration gradually increases from 9 to 23 months of age (with slight changes until 30 months) in Japanese Black steer^29,30^. Additionally, leptin level changes with the body fat mass and stimulation of insulin^31^. Thus, *LEPR* regulation in the late fattening phases may be attributed to fat accumulation and increased insulin secretion, and the increased leptin levels may lead to a decrease in leptin receptor levels. The insulin receptor plays a key role in glucose homeostasis and is closely regulated by hormones such as insulin, IGF-I, and IGF-II. Fisher and Zhang^32,33^ reported that plasma insulin levels were elevated in liver insulin receptor knockout mice, and the insulin regulation of receptors was related to the inhibition of glycogenolysis and gluconeogenesis. Previous studies in cattle have reported that the mRNA expression of insulin receptor in hepatocytes decreased with increasing insulin concentration in a dose-dependent manner^33^, and that dairy cattle with ketosis and fatty liver showed low mRNA expression of hepatic insulin receptor^34^. FOXO3 is also inactivated by the binding of insulin and its receptor, regulates the expression of gluconeogenesis-related genes, and promotes gluconeogenesis. The hepatic suppression of *FOXO1* and *FOXO3* causes hypoglycemia and hyperlipidemia in *FOXO1* and *FOXO3* knockout mice; moreover, the expression of hepatic *FOXOs* affects the reduction of gluconeogenic gene expression and insulin resistance^35^. Additionally, FOXO3 also activates the SESN3, which seems to be related the reactive oxygen species rescue pathway^36^.

We also found that the expression of genes related to lipid metabolism such as fatty acid-binding protein 4 (*FABP4*) was increased in late fattening phases**,** whereas fatty acid desaturase 1 (*FADS1*), *FADS2*, and stearoyl-CoA desaturase (*SCD*) expression was decreased. *FABP4* encodes the fatty acid binding protein, which is considered as related to the uptake and metabolism of fatty acids. In ruminants, FABP4 shows activity in the longissimus dorsi muscle; however, because of its low abundance, its expression and function have mainly studied in the adipose tissue^37^. The *FABP4* genotype significantly affects the marbling score in Wagyu and Limousin crossbred cattle^38^; FABP4 may be associated with the fatty acid composition of intramuscular fat tissue^39^. Here, *FABP4* expression was higher in the late fattening phases than in the early phases. This increase in gene expression related to fat absorption and metabolism is thought to reflect the active accumulation of fat or other energy source in the liver during the late fattening phases. FADS1 and FADS2 are associated with the composition of fatty acids such as arachidonic (C20:4n-6), linoleic (C18:2n-6), alpha-linolenic (C18:3n-3) and eicosadienoic acids^40^. Supplementation of dietary fat in lambs results in increased expression levels of hepatic *FADS1*, along with increased intramuscular docosahexaenoic acid (DHA) levels in the DHA supplemented group^41^. Therefore, the changes in *FADS1* and *FADS2* expression may be related to changes in the fat composition with changes in feed. *SCD* gene expression is related to the oleic acid content in the intramuscular and perinephric adipose tissues of Japanese Black cattle^42^. The percentage of oleic acid generally increases with fattening period^43^. However, we found that the hepatic *SCD* expression decreased in late fattening phases (Supplementary Table S7 online), similar to *FADS1* and *FADS2* gene expressions. The effect of the fattening period on *SCD* expression seems to differ across tissue type, and the regulatory mechanism in the liver requires further analysis.

To identify the significant pathways involving the DEGs between the early and late fattening phases, we performed the KEGG pathway analysis. In total, 33 pathways were significantly enriched for the identified DEGs. The pathway analysis suggested that the main physiological changes associated with fattening period were related to energy and lipid metabolism in Japanese Black steers.

### Blood metabolic profiles and liver transcriptomes related with carcass traits in Japanese Black cattle

Here, blood triglyceride contents were significantly positively correlation with rib eye area and BMS in T1. Blood triglyceride levels are negatively correlated with the level of intramuscular fat accumulation^18^ which may explain the inconsistent results in prior studies. In our study, the blood ALT level was positively correlated with carcass traits in T1, and the ALT concentration was higher in steers with high carcass weight than in steers with relatively low carcass weights in all fattening periods. This indicates that lipid accumulation with changing fattening diets may contribute to carcass traits such as BMS and carcass weight.

The blood aspartic acid had a positive correlation with rib thickness and BMS, and α-amino adipic acid, 1-methylhistidine, and hydroxyproline showed a strong positive correlation with rib thickness in T1. A study on the Wagyu crossbred reported that aspartic acid showed positive correlation with growth rate, no significant correlation with other properties, and that methyl histidine showed a negative correlation with the rump fat^44^. Lee et al.^45^ reported that α-amino adipic acid is correlated with adipogenic differentiation, and that the α-amino adipic acid levels were increased in cell and mouse models of obesity-related insulin resistance. Here, the blood α-amino adipic acid sharply increased between T1 and T2, corresponding to the changes in insulin levels. Thus, the correlation with carcass traits appears to be similar to that with insulin, likely because the blood α-amino adipic acid level reflects changes in insulin levels.

Interestingly, our results showed that ketogenic amino acids such as isoleucine, leucine, tyrosine, and phenylalanine were negatively correlated with most carcass traits in early fattening phases. Ketones were used as an energy source in early fattening phases and more amino acids were consumed, which may have led to a negative relationship between the blood amino acids and the carcass traits of Japanese Black steers. This result suggested that Japanese Black steers in the early fattening phases consumed a lot of amino acids to replenish the considerable amount of energy needed through ketones, resulting in a negative correlation between amino acids and productivity.

In the late fattening phases, glucose had a strong positive correlation with subcutaneous fat. Similar to the results of the present study, some previous studies have reported that glucose level is positively correlated to rump fat and glucose in Wagyu crossbred steers^44^. Glucose, along with acetate^46^ is known as the precursor to fatty acid biosynthesis in ruminants, and blood glucose level is deeply related to the accumulation of intramuscular and subcutaneous fat^10^. Here, the decreased glucose level in late fattening phases may have been to be due to the increase in intracellular influx of glucose by insulin. The positive correlation between blood glucose and subcutaneous fat suggested that more fat is accumulated in Japanese Black cattle with high blood glucose levels. Blood insulin showed a strong positive relationship with carcass weight, growth rates, rib thickness, and BMS. The steers with high carcass weight and BMS had higher concentrations of insulin compared to those with low carcass weight and BMS. As noted in an earlier study, insulin promotes the intracellular influx of glucose for fat accumulation, and blood insulin concentrations are associated with carcass traits such as carcass weight, growth rate, rib thickness, and BMS^10,18^. This suggests that insulin is a more important regulator of lipid metabolism in the T3 phase than in the T1 phase in Japanese Black steers. We also investigate hepatic DEGs according to carcass weight (High vs Low) and BMS (High vs Low) to investigate the physiological changes with productivity in Japanese Black cattle. In total, 14 genes were differentially expressed when compared between carcass weights (High vs Low) and were mainly involved in organ development, cell growth, and muscle contraction. In comparison between BMS (High vs Low), 5 genes related to calcium channel and cytoskeleton components were found. This study revealed slight changes in the liver transcriptome according to carcass characteristics. No differences in energy- and fat metabolism-related genes were found in each fattening period, likely due to long-term adaptation and small metabolic changes into 2 different carcass weight and BMS categories. However, we found differences in the candidate genes and pathways responsible with large metabolic changes between the T1 and T3 phases.

The results suggest that the main physiological difference in Japanese Black cattle is that the high-production steers show high blood insulin concentration. As with high insulin levels in the late fattening phases, the steers that consumed a lot of high-energy diets had high energy intake compared to other steers with relatively low intake, resulting in the secretion of insulin to store excess energy.

## Conclusion

Our study showed that the physiological changes associated with fattening phases were typically characterized by high-energy diets for growth and carcass traits in Japanese Black steers. The increased glucose, total cholesterol, and NEFA and decreased ketones during the fattening period were related to lipid metabolism, fat accumulation, and fatty acid synthesis, and strongly associated to insulin regulation. Additionally, the DEGs—such *as INSR, LEPR, FOXO3, FABP4, FADS1*, and *FADS2*—enriched in relation to lipid metabolism were regulated to accumulate energy in the liver; insulin may have affected this process. Insulin also showed differences depending on the productivity of cattle. Therefore, the physiological characteristics of Japanese Black cattle are as follows: the physiological changes during the fattening periods result from changes in energy content and composition of diets; the changes in liver metabolism according to fattening period are strongly related to aspects of fat metabolism, such as fat accumulation and transport; and insulin regulates physiological changes according to fattening periods and productivity. Our results also suggest that the above blood metabolites and liver transcriptome could be used as parameters to determine the metabolic status of Japanese Black steer bred and raised in accordance with the typical feeding systems in Japan. Additionally, the parameters could be used to evaluate metabolic status in other cattle breeds for beef production.

## Methods

### Animals and experimental design

The animal experiments were performed at the Hyogo Prefectural Technology Center of Agriculture, Forestry and Fisheries (Hyogo Prefecture, Japan) and conducted by the Guideline for the Institute of Livestock and Grassland Science^47^ and the Ethical guidance of the Hyogo Prefectural Institute of Agriculture of Forestry and Fisheries Animal Care and Use Committee. Experiment protocol was evaluated and approved by Hyogo Prefectural Institute of Agriculture of Forestry and Fisheries Animal Care and Use Committee (approval number: H2018-01). All animal experiments were carried out in accordance with ARRIVE guidelines (https://arriveguidelines.org).

Twenty-one Japanese Black steers aged 12 months (initial body weight, 335.6 ± 19.8 kg) were reared until 30 months of age (final body weight, 742.1 ± 49.9 kg). The experimental period was divided into early fattening (12–14 months of age; T1), middle fattening (15–22 months of age; T2), and late fattening phases (23–30 months of age; T3). Experimental animals were fed concentrate and roughage (rice straw and kraft pulp feed) twice daily (at 09:30 and 15:00). Water was always freely available, and other feeding management was conducted in accordance with the practices of the Hyogo Prefectural Technology Center. The steers were fed with specific amounts of formula diet that were changed each fattening period as shown in Table 5. The chemical composition of the formula diet was analyzed according to standard tables of feed composition in Japan^48^ and provisional values for official specification set by the Ministry of Agriculture, Forestry and Fisheries^49^. The adequacy rate of the diet was calculated based on the nutrient requirement of Japanese Feeding Standard for Beef cattle^50^. After the feeding experiment, four cattle from each group with the highest and lowest productivity (carcass weight and BMS) were chosen, and changes in the physiological parameters and liver transcriptome of Japanese Black steers were evaluated.

### Sample preparation and carcass trait assessment

Experimental samples, including blood and rumen fluid, were collected at the early fattening (13 months of age), middle fattening (20 months of age), and late fattening phases (28 months of age) from 21 Japanese Black steers. Blood samples were collected at 13:00, 3 h after morning feeding from the jugular vein into heparin-sodium tubes (Venoject II VP-H100K, Terumo, Tokyo, Japan). All samples were transferred to the laboratory on ice and stored until centrifugation at 3000×*g* for 15 min. Plasma samples were stored at − 30 °C until metabolic profiling.

Rumen fluid was collected using a suitable catheter. Approximately 200 mL of rumen fluid was collected in a sterilized and dried flask and immediately filtered through 4 layers of cheesecloth to separate the rumen fluid from the diet particles. The pH of rumen samples was measured using a digital electric pH meter after filtering the rumen fluid. The filtrates were stored at − 80 °C until component analysis.

Liver tissue was biopsied for 20, 20, and 19 heads from T1, T2, and T3, respectively, as described previously^51^. The biopsy site was at the intersection of the right 11th to 12th intercostal space and the line from the right point of the acromion to the tuber coxa. The biopsy site was anesthetized with xylazine. A 0.5 cm long stab incision was made in the skin and the biopsy was performed using an automatic biopsy gun with 150 mm biopsy needles (ACECUT ACE-141502, DELTA surgical, Staffordshire, UK). After the biopsy, the spot was disinfected with 10% isodine gel. All tissues were rapidly separated, immediately frozen in liquid nitrogen, and stored at − 80 °C until RNA extraction.

All steers were slaughtered at a commercial meat abattoir after a 24 h rest period. Carcasses were chilled for 24 h at 0 °C, after which the left side was opened between the 6th and 7th ribs to evaluate the yield and quality of the carcass according to the standard criteria of the Japan Meat Grading Association^7^. The traits measured during carcass evaluation were carcass weight, beef marbling score (BMS), rib eye area, rib thickness, and subcutaneous fat thickness.

### Blood metabolome and rumen fermentation profiling

Blood metabolites were analyzed using an Automatic Biochemical Analyzer (HITACHI 7070, Hitachi, Ltd., Tokyo, Japan). The analyses included measurements of total protein, albumin, blood urea nitrogen (BUN), creatinine, total cholesterol, triglycerides, NEFA, glucose, ALP, AST, ALT, LD, γ-GTP, creatine kinase, acetate, BHB, and total ketone bodies. Plasma insulin, IGH-1, and cortisol were assayed by enzyme immunoassay according to manufacturer instructions using a multi-species insulin ELISA kit (Mercodia bovine insulin ELISA, Mercodia AB, Uppsala, Sweden), human IGF-1 ELISA kit (Human IGF-I, R&D system, Minneapolis, USA), and cortisol ELISA kit (Cortisol ELISA kit, Enzo Life Sciences Inc., Budapest, Hungary), respectively.

After adding trichloroacetic acid to the plasma and filtering the proteins through a membrane filter, the blood amino acid concentrations were determined by a high-speed amino acid spectrometer (L-8900, Hitachi High Tech, Tokyo, Japan). Total VFA and VFA components (acetic acid, propionic acid, butyric acid, and valeric acid) were separated and quantified by gas chromatography (GC2014, Shimadzu, Kyoto, Japan) using a packed glass column (Thermon-3000 [3%]) on a Shimalite TPA 60–80 support (Shinwa Chemical Industries Ltd., Kyoto, Japan). The operating conditions of gas chromatography were as follows: career gas, N2; flow volume, 30 mL/min; temperature of the column injection and FID detection, 220 °C; and temperature of the column oven, 140 °C. The ammonium nitrogen concentration in the rumen fluid was quantified by the steam distillation method using an automatic nitrogen analyzer (Kjeltec Auto 1,035, Tecator, Sweden).

### RNA purification

The 20 (T1), 20 (T2), and, 19 (T3) liver tissue samples were used for RNA-Seq. Liver tissues were soaked in 200 μL RNAiso Plus (TAKARA Bio Inc., Shiga, Japan) in liquid nitrogen and homogenized using a Multibeads shocker (YASUIKIKAI Inc., Osaka, Japan) following manufacturer instructions. Homogenization was carried out twice at 2000 rpm for 10–15 s, and 800 μL RNAiso was added at 25 °C. The homogenate was collected in 1.5 mL tubes, 200 μL chloroform was added to it, and the solution was thoroughly mixed. After settling for 5 min at room temperature (25 °C), the solution was centrifuged at 12,000 ×*g* for 15 min at 4 °C. The supernatant was collected and mixed with 500 μL isopropanol, and the mixture was centrifuged at 12,000×*g* for 15 min at 4 °C to precipitate the RNA. The supernatant was carefully removed. The pellet was washed twice with 75% cold ethanol and dissolved it with RNase-free water. Purified total RNA was quantified using a Nano Drop ND-1000 Spectrophotometer V3.7.1 (Thermo Fisher Scientific, Waltham, MA, USA) at wavelengths of 230, 260, and 280 nm. The purity of total RNA was determined as the A_260_/A_280_ ratio (expected values > 1.8) and verified by 1.0% agarose gels electrophoresis. All samples were stored at − 80 °C.

### RNA-Seq and data analyses

To determine whether the purified total RNA could be used in RNA-seq, the RNA integrity number (RIN) was confirmed in a Tape Station 4200 using an RNA Screen Tape kit (Agilent Technologies, Santa Clara, CA, USA). The average RNA integrity number and mRNA rate were 7.1 (range, 6.9–7.9) and 0.8 (range, 0.7–1.2), respectively. RNA-seq libraries were prepared using a TruSeq stranded mRNA Kit (Illumina, San Diego, CA, USA) and sequenced using a NovaSeq 6000 platform at Macrogen Japan Corporation. The quality of the sequencing reads was evaluated using FastQC software (http://www.bioinformatics.babraham.ac.uk/projects/fastqc, version 0.11.8). Sequencing reads were trimmed using Trim Galore software (http://www.bioinformatics.babraham.ac.uk/projects/trim_galore/, version 0.5.0) and reassessing using FastQC software. Sequencing reads were mapped to the ARS-UCD1.2 bovine reference genome (ftp://ftp.ensembl.org/pub/release-101/fasta/bos_taurus/dna/) using the HISAT2 software^52^. String Tie software^52^ was used to calculate the read counts for expressed transcripts with the GTF Bovine gene annotation file (ftp://ftp.ensembl.org/pub/release-101/gtf/bos_taurus). Normalization of counts and PCA were performed using the DESseq2 package^53^ in R statistical software to determine the relationship among individual samples. Two samples from T2 were identified as outliers and removed, because they were out of the cluster in the PCA score plot. Thereafter, 20 (T1), 18 (T2), and 19 (T3) samples were used for subsequent data analyses. The DESeq2 package^53^ was also used to detect the DEGs between 2 groups (T1 vs T2, T2 vs T3, and T1 vs T3) by normalizing the counts and using Wald test. FDR was calculated to account for multiple testing. Significantly DEGs were defined as having an FDR < 0.05 and |log_2_ fold change|> 1.5. Functional annotation enrichment analyses using the GO^54,55^ and KEGG databases^56^ were performed using the Database for Annotation, Visualization, and Integrated Discovery (https://david.ncifcrf.gov/summary.jsp). GO terms and KEGG pathways with *P*-values ≤ 0.05 were considered as significantly enriched.

### Statistical analysis of physiological parameters

Statistical analyses were performed using SAS 9.4 software (2012 SAS Institute, Inc., Cary, NC, USA) and R 4.0.3 software (2020, R Foundation for Statistical Computing, Vienna, Austria). The significant differences in physiological parameters, including the blood metabolites, blood amino acid, hormones, and rumen fermentation products, among fattening phases was evaluated with mixed model analysis of variance (ANOVA) with the fixed effect of period (T1, T2, and T3) and random effect of animal. Following mixed model analysis, post-hoc multiple comparisons among fattening periods were performed using Tukey–Kramer method. PCAs were performed using the relative concentrations of identified blood metabolites and rumen fermentation characteristics as variables in the model with all possible PCs. PC1 and PC2 score plots were prepared and the data points were colored to visualize the potential clustering of Japanese Black steers according to their fattening phases. From the data of each fattening period, correlation coefficients between the blood parameters (or rumen fluid parameters) and carcass traits were analyzed using Pearson’s correlation. To investigate the associations between physiological differences and productivity in Japanese Black cattle, the significant differences in physiological parameters were analyzed using mixed model ANOVA with the random effect of animal and fixed effects of the fattening period (T1, T2, and T3), productivity (carcass weight: high vs low, BMS: high vs low), and fattening period × productivity. Significant differences in carcass traits according to productivity were calculated using ANOVA and Kruskal Wallis test for data meted normal distribution and for those did not, respectively. In the present study, differences were considered as significant at *P* < 0.05 and statistical tendencies were indicated as 0.05 < *P* ≤ 0.10.

## Supplementary Information

Supplementary Legends.

Supplementary Figure 1.

Supplementary Figure 2.

Supplementary Table 1.

Supplementary Table 2.

Supplementary Table 3.

Supplementary Table 4.

Supplementary Table 5.

Supplementary Table 6.

Supplementary Table 7.

Supplementary Table 8.

Supplementary Table 9.

Supplementary Table 10.

Supplementary Table 11.

Supplementary Table 12.

Supplementary Table 13.

Supplementary Table 14.

Supplementary Table 15.

Supplementary Table 16.

Supplementary Table 17.

## Data Availability

RNA sequence data generated from the present study have been submitted to NCBI BioProject database under project number PRJNA760255. The data will be available with the following link on May 31, 2022: https://www.ncbi.nlm.nih.gov/sra/PRJNA760255.
