# Supplementary material for: Changes in the liver transcriptome and physiological parameters of Japanese Black steers during the fattening period
Source: Sci Rep. 2022 Mar 7;12:4029. doi: 10.1038/s41598-022-08057-8 (PMC8901683; doi:10.1038/s41598-022-08057-8)

Changes in the liver transcriptome and physiological parameters of Japanese Black steers during the fattening period

Minji Kim, Tatsunori Masaki, Kentaro Ikuta, Eiji Iwamoto, Yoshinobu Uemoto, Fuminori Terada, and Sanggun Roh

Supplementary Table legends;

Supplementary Table S1. Concentrations of blood amino acid during the early (T1; 13 months of age), middle (T2; 20 months of age), and late fattening phases (T3; 28 months of age) in Japanese black cattle. Values indicate mean and means in the same row with different superscripts differ significantly (*P* < 0.05).

**Supplementary Table S2.** **Composition of rumen fluid during the early (T1; 13 months of age), middle (T2; 20 months of age), and late fattening phases (T3; 28 months of age) in Japanese black cattle.** Values indicate mean and means in the same row with different superscripts differ significantly (*P* < 0.05).

**Supplementary Table S3.** **Statistics and mapping results of RNA-seq data for early (T1; 13 months of age), middle (T2; 20 months of age), and late fattening phases (T3; 28 months of age).** GC: guanine and cytosine, AT: adenine and thymine, Q20: ratio of bases that have phred quality score > 20, Q30: ratio of bases that have phred quality score > 30.

**Supplementary Table S4.** **Statistics and mapping results of RNA-seq data for each Japanese Black cattle.**  T1: early fattening phases (13 months of age), T2: middle fattening phases (20 months of age), T3: late fattening phases (28 months of age). GC: guanine and cytosine, AT: adenine and thymine, DQ20: ratio of bases that have phred quality score > 20, Q30: ratio of bases that have phred quality score > 30.

**Supplementary Table S5. List of differentially expressed genes in carcass weight groups (High vs Low) in each fattening period.** T1: early fattening phases (13 months of age), T2: middle fattening phases (20 months of age), T3: late fattening phases (28 months of age).

**Supplementary Table S6**. **List of differentially expressed genes in beef marbling score groups (High vs Low) in each fattening period.** T1: early fattening phases (13 months of age), T2: middle fattening phases (20 months of age), T3: late fattening phases (28 months of age).

**Supplementary Table S7.** **List of differentially expressed genes in T1 (13 months of age) vs T3 (28 months of age) groups.**

**Supplementary Table S8. Gene ontology of differentially expressed genes in T1 (13 months of age) vs T3 (28 months of age) groups.**

**Supplementary Table S9. KEGG pathways of differentially expressed genes in T1 (13 months of age) vs T3 (28 months of age) groups.**

**Supplementary Table S10. Carcass traits of Japanese Black cattle used in present study (n=21).**

**Supplementary Table S11. Heat map illustrating the correlations between physiological parameters and carcass traits in the middle fattening period (20 months of age).** BMS: beef marbling score, BUN: blood urea nitrogen, NEFA: non-esterified fatty acid, ALP: alkaline phosphatase, AST: aspartate aminotransferase, ALT: alanine aminotransferase, γ-GTP: gamma(γ)-glutamyl transferase, LD: lactate dehydrogenase, CK: creatine kinase, BHBA: β-hydroxybutyric acid, IGF-I: insulin-like growth factor 1. ** and * indicate *P* < 0.01 and *P* < 0.05, respectively.

**Supplementary Table S12. Concentrations of blood metabolites and hormones in carcass weight groups (High vs Low).** Carcass weights: High (n=4) and Low (n=4). BUN: blood urea nitrogen, NEFA: non-esterified fatty acid, ALP: alkaline phosphatase, AST: aspartate aminotransferase, ALT: alanine aminotransferase, γ-GTP: gamma(γ)-glutamyl transferase, LD: lactate dehydrogenase, CK: creatine kinase, BHBA: β-hydroxybutyric acid, IGF-I: insulin-like growth factor 1. Values indicate mean.

**Supplementary Table S13. Concentrations of blood amino acids in carcass weight groups (High vs Low).** Carcass weights: High (n=4) and Low (n=4). Values indicate mean.

**Supplementary Table S14.** **Composition of rumen fluids in carcass weight groups (High vs Low).** Carcass weights: High (n=4) and Low (n=4). Values indicate mean.

**Supplementary Table S15. Concentrations of blood metabolites and hormones in beef marbling score (BMS) groups (High vs Low).** BMS groups: High (n=4) and Low (n=4). BUN: blood urea nitrogen, NEFA: non-esterified fatty acid, ALP: alkaline phosphatase, AST: aspartate aminotransferase, ALT: alanine aminotransferase, γ-GTP: gamma(γ)-glutamyl transferase, LD: lactate dehydrogenase, CK: creatine kinase, BHBA: β-hydroxybutyric acid, IGF-I: insulin-like growth factor 1. Values indicate mean.

**Supplementary Table S16. Concentrations of blood amino acids in beef marbling score (BMS) groups (High vs Low).** BMS groups: High (n=4) and Low (n=4). Values indicate mean.

**Supplementary Table S17.** **Composition of rumen fluid in beef marbling score (BMS) groups (High vs Low).** BMS groups: High (n=4) and Low (n=4). Values indicate mean.

**Supplementary Figure legends;**

**Supplementary Figure S1. RNA-Seq analyses of differentially expressed genes of Japanese black cattle in the early (T1; 13 months of age), middle (T2; 20 months of age), and late fattening phases (T3; 28 months of age).**

**Supplementary Figure S2. Volcano plots of differentially expressed genes in early (T1; 13** **months of age) vs middle (T2; 20 months of age) vs late fattening phases (T3; 28 months of age).** *P* < 0.05, log_2_FC > ±1.5, and base mean > 30.


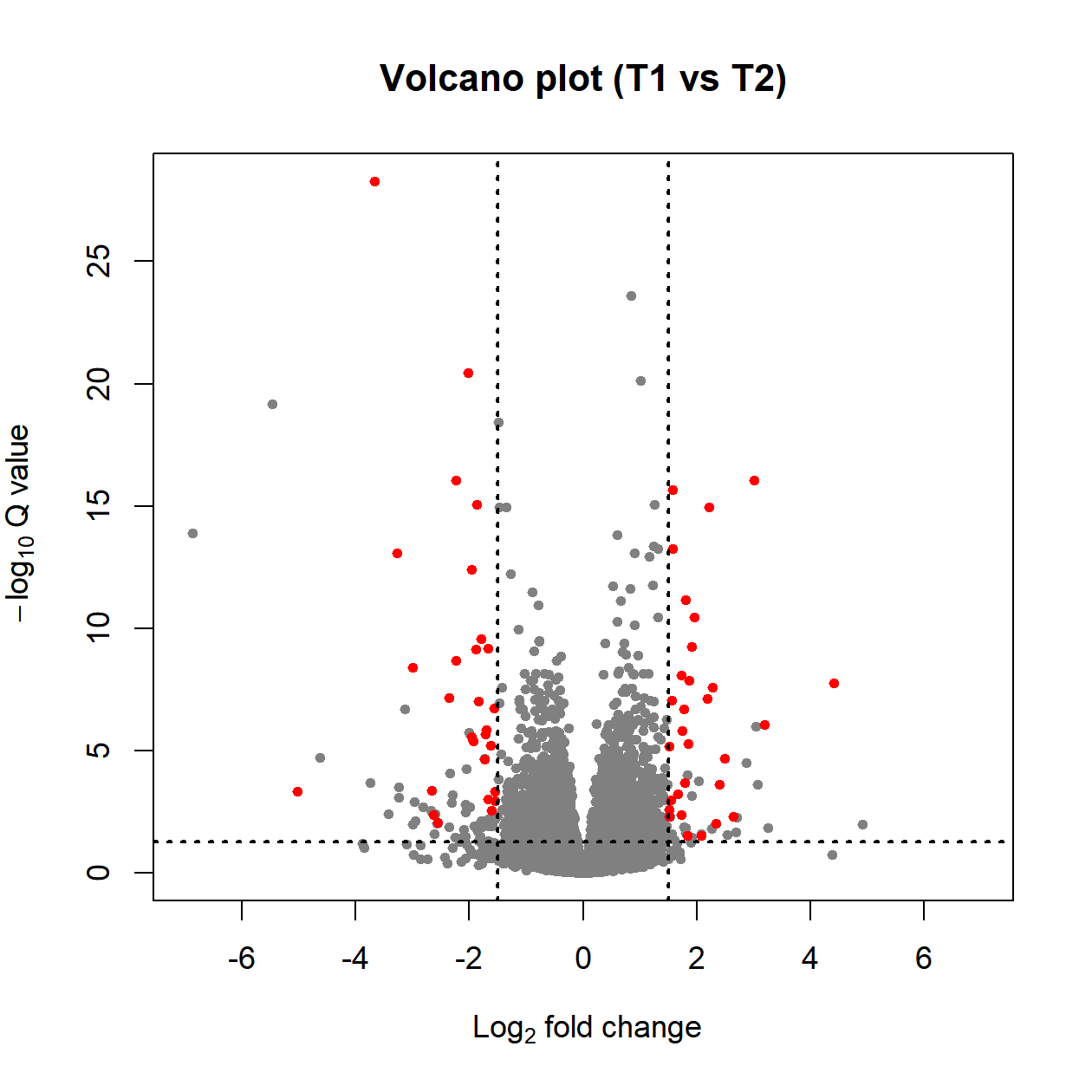

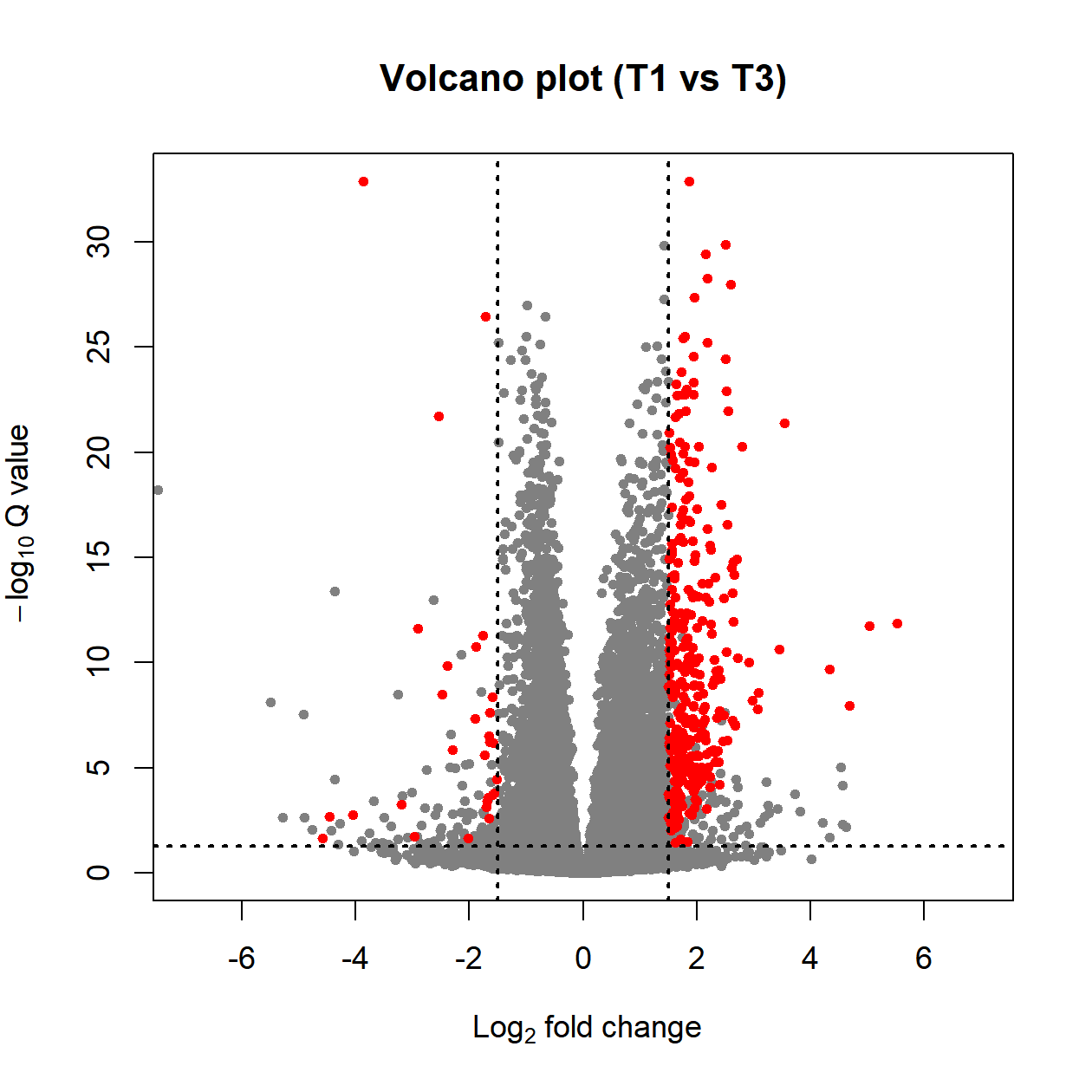

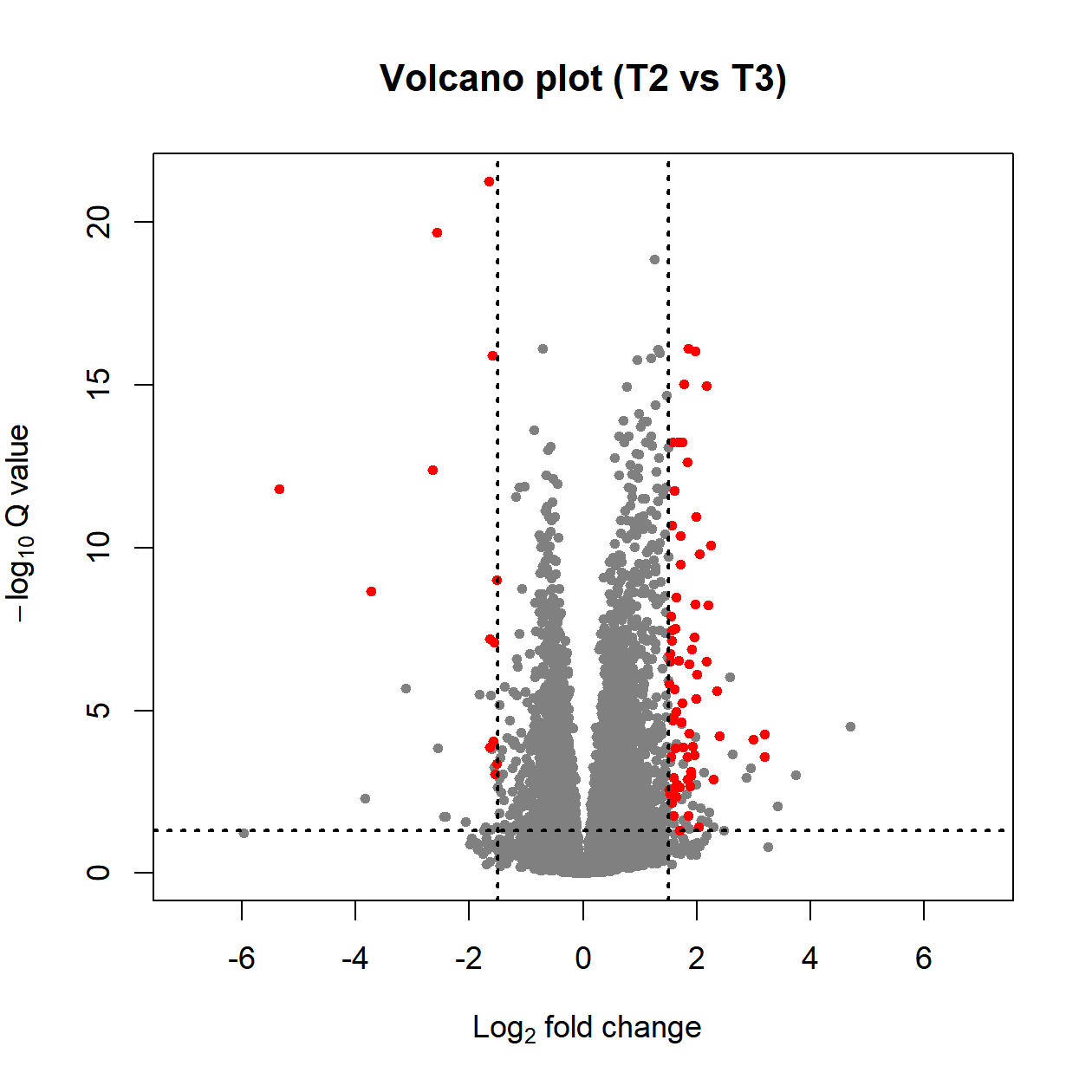

Supplement: Supplementary file 1 — Supplementary Legends. [file 41598_2022_8057_MOESM1_ESM.docx]
