# Supplementary figures and images for: Changes in the liver transcriptome and physiological parameters of Japanese Black steers during the fattening period
Source: Sci Rep. 2022 Mar 7;12:4029. doi: 10.1038/s41598-022-08057-8 (PMC8901683; doi:10.1038/s41598-022-08057-8)

Supplementary Figure S1

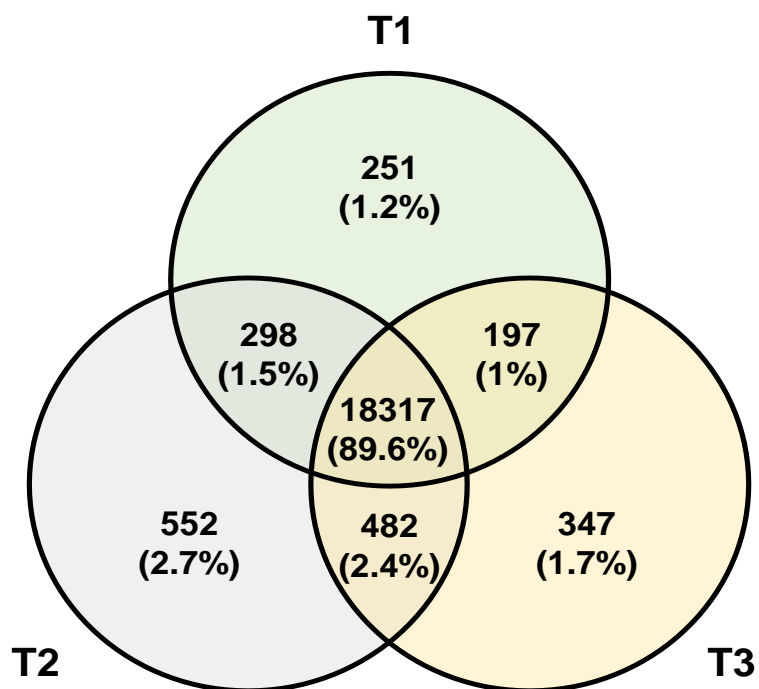

Supplement: Supplementary file 2 — Supplementary Figure 1. [file 41598_2022_8057_MOESM2_ESM.pdf]

Supplementary Figure S2

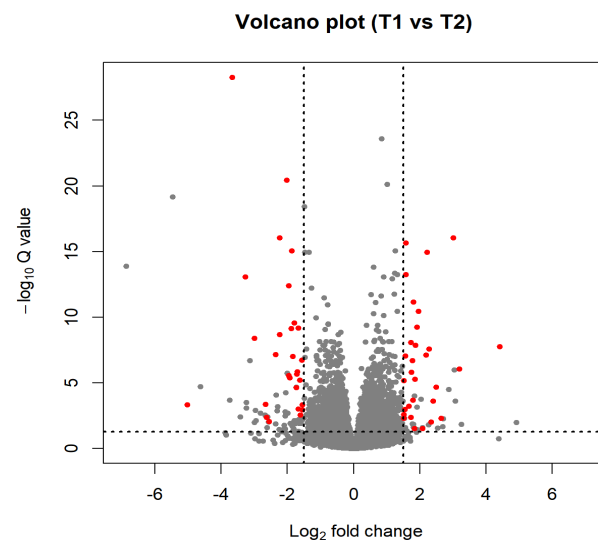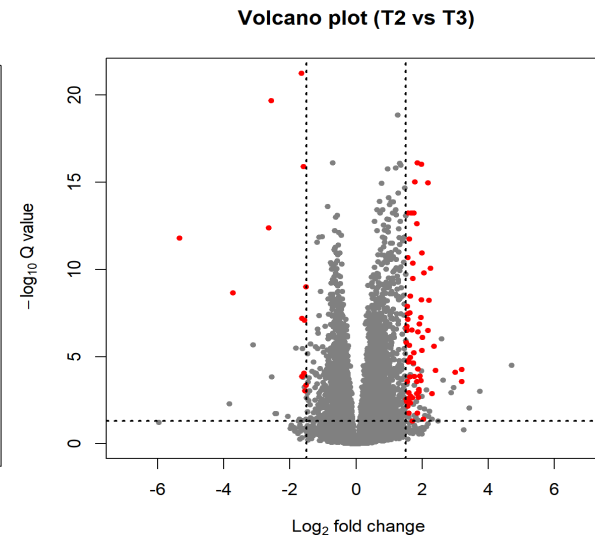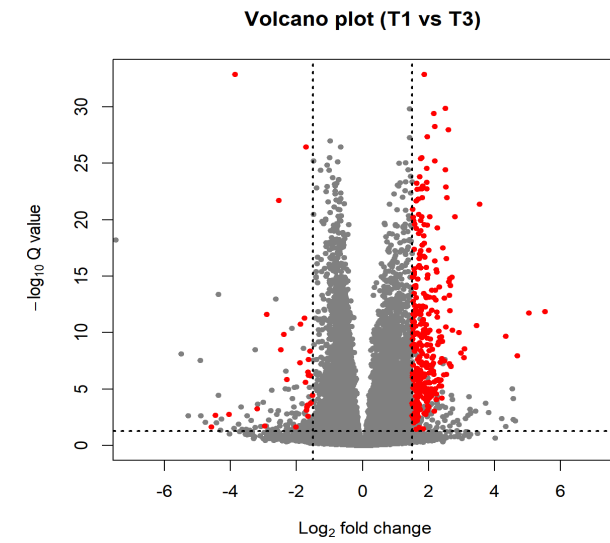

Supplement: Supplementary file 3 — Supplementary Figure 2. [file 41598_2022_8057_MOESM3_ESM.pdf]
